# Anthropometric Measures of Obesity and Food Consumption Among U.S. Chinese Older Adults

**DOI:** 10.1093/geroni/igaa057.768

**Published:** 2020-12-16

**Authors:** Shenglin Zheng, Qun Le, XinQi Dong

**Affiliations:** Rutgers University, New Brunswick, New Jersey, United States

## Abstract

Most research uses body mass index (BMI) alone to measure obesity. Combined with waist circumference (WC), BMI may better identify obesity-related health risk. And diet is a key component of obesity management. To better understand the relationship between obesity and diet, this study aims to examine two anthropometric measures of obesity and the food consumption among U.S. Chinese older adults. Data were drawn from the PINE study wave III (2015-2017), a prospective cohort study of community-dwelling Chinese older adults (N=3053). We categorized participants into 6 groups: normal BMI (18.5-24.9) with normal WC (women ≤ 88cm and men ≤102cm), normal BMI with high WC (women WC >88cm and men WC >102cm), overweight (BMI=25.0-29.9) with normal WC, overweight with high WC, obese (BMI >30) with normal WC, and obese with high WC. A forty-eight-item food frequency questionnaire was used to measure frequencies of vegetables, fruits, grains, protein foods, dairy, sweets, and alcohol intake. Almost 12% participants had normal BMI but high WC and 10% were overweight with high WC. Participants who were overweight with high WC reported the highest intake of vegetables among groups. Participants with higher WC had significantly higher fruit consumption, compared to those with normal WC, regardless of their BMI. Spearman correlation analysis showed that being overweight with a high WC was correlated with higher frequencies of vegetables and fruits intake and having normal BMI with normal WC was correlated with higher alcohol intake. The findings provide new insights for future research and interventions on obesity/chronic disease management.

